# Precocious puberty in patients with Pompe disease

**DOI:** 10.3389/fendo.2023.1150498

**Published:** 2023-08-15

**Authors:** Meng-Ju Melody Tsai, Mei-Huei Chen, Yin-Hsiu Chien, Yi-Ching Tung

**Affiliations:** ^1^ Department of Pediatrics, National Taiwan University Hospital and College of Medicine, National Taiwan University, Taipei, Taiwan; ^2^ Department of Pediatrics, National Taiwan University Hospital Yunlin Branch, Yunlin, Taiwan; ^3^ Institute of Population Health Sciences, National Health Research Institutes, Miaoli, Taiwan; ^4^ Department of Medical Genetics, National Taiwan University Hospital, Taipei, Taiwan

**Keywords:** Pompe disease, precocious puberty, phthalate exposure, enzyme replacement therapy, mono-2-ethylhexyl phthalate

## Abstract

**Introduction:**

The life expectancy of Pompe disease patients has increased due to improved neonatal screening and enzyme replacement therapy. Nevertheless, the potential effect of frequent medical device exposure on pubertal development in these patients is not well understood, so further investigation is warranted.

**Methods:**

In this cross-sectional study, we assessed the growth and puberty of nine Pompe disease patients. In addition, to determine the effects of frequent plastic medical device exposure in these patients, we measured urinary phthalate metabolites before and one day after enzyme replacement therapy.

**Results:**

Five out of nine patients (55%) with Pompe disease on enzyme replacement therapy had precocious puberty. Patients with precocious puberty had significantly shorter predicted adult heights compared to those with normal puberty (*p* = 0.014). The levels of mono-2-ethylhexyl phthalate (MEHP) and mono(2-ethyl-5-carboxypentyl) phthalate (MECPP) increased after enzyme replacement therapy, but the average levels of phthalate metabolites did not significantly differ between patients with normal and precocious puberty.

**Conclusion:**

Pompe disease patients on enzyme replacement therapy tend to have precocious puberty, which may reduce their adult height. There are no significant differences in urinary phthalate metabolites between normal and precocious puberty patients. Regular follow-up of growth and puberty in Pompe disease patients is important to improve their health outcomes.

## Introduction

Pompe disease results from the deficiency of acid α-glucosidase (GAA), which leads to lysosomal accumulation of glycogen in various organs, especially cardiac and skeletal muscles (1). In the classical infantile-onset Pompe disease (IOPD), symptoms onset at a median age of 2 months and, if untreated, death occurs at a median age of 8.7 months (2). The concept of ERT was first developed in 1964 by Christian de Duve and Roscoe Brady. It was not implemented in clinical practice till 1991, when the FDA granted orphan drug approval for the treatment of Gaucher disease (3). The first ERT for Pompe disease was available in 2006 (4). Currently, enzyme replacement therapy (ERT) with human recombinant GAA is the only approved therapy to reduce glycogen storage in muscles. Newborn screening for Pompe disease was first introduced in Taiwan in 2005 (5, 6). Earlier treatment of IOPD patients is associated with better outcomes (7). However, new medical problems, related to both the treatment and disease, have emerged among long-term survivors, such as hypernasality, residual muscle weakness, and brain signal changes. Patients who require frequent ERT or intravenous infusion often encounter complicated medical issues which might distract them from the pubertal timing.

The growth and puberty of long-term survivors of Pompe disease have not been thoroughly evaluated. About 19.5% of IOPD patients on ERT had premature pubarche (8); however, no studies on gonadarche have been conducted. Although the etiology of early adrenal activation in these patients is unclear, environmental factors should be considered. Endocrine-disrupting chemicals (EDCs), such as phthalates and bisphenol A, may interfere with the homeostasis of hormones, particularly in the reproductive system (9). Plastic indwelling medical devices are a source of iatrogenic exposure to these EDCs. Compared to adults, children are much more vulnerable and sensitive to phthalate exposure (9, 10). Some studies have reported increased phthalate metabolite levels in girls with premature thelarche and precocious puberty (11, 12). Among Pompe disease patients on regular ERT for several years, the effects of exposure to phthalates via medical devices on earlier puberty onset remain unclear. Considering the potential for phthalate exposure from medical devices during regular weekly/biweekly ERT, we analyzed seven urinary phthalate metabolites before and after ERT administration. We are the first report to demonstrate the growth and puberty of Pompe disease patients and evaluate their phthalate metabolite levels.

## Materials and methods

### Patients

In this study, we included Pompe disease patients who were older than 9 years in boys and 8 years in girls at the end of the analysis. A total 20 patients with Pompe disease were enrolled. All participants underwent ERT and had regular follow-ups at the National Taiwan University Hospital in Taiwan from 2006 to 2021. Among them, seven patients with late-onset Pompe disease were excluded because they received ERT after puberty onset. Three patients with incomplete follow-up and another one of serious illness rendering him bedridden were also excluded. Finally, nine patients (five males and four females) with IOPD were eligible in the analysis; two of them were atypical IOPD. The characteristics of these patients have been described previously (13). The patients underwent detailed physical examination, including assessment of height, weight, and pubertal signs. The height, weight, and body mass index (BMI) were compared with the normal Taiwanese population and presented as standard deviation score (SDS) (14). The age of puberty onset was defined as the time of the following criteria: (i) presence of secondary sexual characteristics, (ii) the growth velocity > 6cm/year, and/or (iii) basal LH level > 0.3 mIU/L and detectable basal E2 (15, 16). Precocious puberty was defined as the puberty onset age before 9 years in boys and 8 years in girls The mid-parental height (MPH) was calculated according to Tanner’s method (17). Bone age was assessed using the Greulich and Pyle method. The predicted adult height (PAH) was calculated according to the Bayley & Pinneau criteria (18). Further, they all completed a questionnaire regarding the use of dietary supplements, traditional Chinese medicine, and skincare products, such as lotions and essential oils, especially the ingredients of lavender or tea tree. The patients were categorized into precocious puberty and normal puberty groups.

### Urine analysis

The first spot urine samples were collected in the early morning on the day and the next day for ERT. Urine samples were stored at −20°C and subjected to ultra-performance liquid chromatography coupled with tandem mass spectrometry (UPLC-MS/MS). The seven phthalate metabolites (mono-methyl phthalate [MMP], mono-ethyl phthalate [MEP], monobutyl phthalate [MBP], mono-benzyl phthalate [MBzP], mono-2-ethylhexyl phthalate [MEHP], mono(2-ethyl-5-carboxypentyl) phthalate [MECPP], and mono(carboxy-isononyl) phthalate [MCiNP]), were measured in the urine, as previously described with some modifications (19, 20). The values of phthalate metabolites (pg/mL) per creatinine (mg/dL) were used to correct the urine dilution for statistical analyses.

### Statistics

The statistical analyses were performed using SPSS software (version 22.0; IBM Corp., Armonk, NY, USA). We compared the auxological data and average urine phthalate metabolites between the normal and precocious puberty groups using the Mann-Whitney U test, and the urine phthalate metabolites before and after ERT using the Wilcoxon signed-rank test. A *p* value < 0.05 was considered statistical significance.

## Results

The auxological data of nine patients are presented in **Table 1**. At the last visit, the median age of patients was 12.9 years old (range: 10.3 to 15.8 years old), with a median height of -0.5 SDS (range: -1.2 to 1.6), a median weight of 0.1 SDS (range: -0.6 to 0.7), and a median BMI of 0.5 SDS (range: -0.8 to 1.7). The patients had received ERT at a dose of 20 mg/kg every 2 weeks to 40 mg/kg every week, since the median age of 0.5 months (range: 0.2 to 37 months). Three girls (75%) and two boys (40%) had precocious puberty. The girl with normal puberty did not experience her menarche by the end of the study, while the other three girls experienced menarche at the ages of 10 years old, 10 years and 8 months old, and 10 years and 11 months old, respectively. On average, it takes 3.0 years from the onset of breast development to menarche. Two patients (No. 2 and 6) attained their adult height. The median adult height (or PAH) of these nine patients was 0.3 SDS (range, -2.4 to 1.7) and −0.5 SDS (range, -2.6 to 2.8) compared to the normal population and their MPH, respectively. All patients had brain magnetic resonance imaging and showed the difference in the degree of hypomyelination over the cortex (21). No lesion was detected over the hypothalamus-pituitary area.

**Table 1:** The puberty evaluation of the 9 Pompe disease patients at the end of the study.

No	Sex	Age of 1^st^ ERT (m)	Motor status	Puberty onset (year)	Age (year)	BA SDS	height SDS	weight SDS	BMI SDS	(P)AH (cm)	(P)AH SDS	(P)AHSDS-MPHSDS
1	F	0.2	Ambulate-ind	7.2	10.8	4.1	0.1	0.1	1.7	147.1	-2.4	-1.8
2	F	0.5	Partial wheelchair-d	7.5	12.9	2.7	-1.2	-0.5	0.2	147.2^a^	-2.4	-2.1^a^
3	F	0.5	Partial wheelchair-d	7.8	10.3	2.4	1.1	0.7	0.5	161.0	0.3	-0.5
4	F	4.5	Ambulate-ind	9.7	11.3	1.2	1.6	-0.2	-0.8	168.1	1.7	2.8
5	M	37.0	Partial wheelchair-d	8.5	14.0	2.5	-1.0	-0.6	0	157.6	-1.9	-2.6
6	M	0.6	Ambulate-ind	8.8	15.2	2.4	-1.2	0.6	1.7	159.2^a^	-1.6	-2.5^a^
7	M	0.4	Partial wheelchair-d	10.7	14.3	0.2	0.0	0.2	0.6	174.6	1.2	-0.2
8	M	0.5	Ambulate-ind	10.7	11	0.3	-0.5	0.1	0.6	172.4	0.8	0.6
9	M	1.0	wheelchair-d	11.3	15.8	0	-0.5	-0.6	-0.3	170.4	0.4	1.3
**Median**		**0.5**		**F: 7.7** **M:10.7**	**12.9**	**2.4**	**-0.5**	**0.1**	**0.5**	**F: 154.1** **M: 170.4**	**0.3**	**-0.48**
**Mean ± SD**		**5.0 ± 12.1**		**F: 8.1 ± 1.1** **M: 10.0 ± 1.3**	**12.8 ± 2.1**	**1.8 ± 1.4**	**-0.2 ± 1.0**	**0.0 ± 0.5**	**0.5 ± 0.8**	**F: 155.9 ± 10.5** **M: 166.8 ± 7.9**	**-0.4 ± 1.6**	**-0.6 ± 1.9**

a Reached adult height; F, female; M, male; ERT, enzyme replacement therapy; d, dependent; ind, independent; BA, bone age; SDS, standard deviation score; BMI, body mass index; (P)AH, (predicted) adult height; MPH, midparental height. The bold values indicate the median and mean values.

**Table 2** compares the precocious (n = 5) and normal puberty (n = 4) groups. The height, weight, BMI, and MPH were similar between the two groups. However, bone age was significantly advanced compared to their chronological age in the precocious compared to the normal puberty group (3.0 SD and 0.4 SD, respectively, *p* = 0.014). After the onset of puberty, the bone age to chronological age (BA/CA) of the precocious puberty patients and the normal puberty patients are not significantly different with an average of 1.2 and 1.3 times, respectively. No patient received gonadotropin-releasing hormone analog (GnRHa). In addition, the adult height (or PAH) SDS were significantly shorter in the precocious than normal puberty group, even when corrected by their MPH (-1.9 and 1.1, respectively, *p* =0.014).

**Table 2:** The comparison of auxological data in the normal puberty and precocious puberty groups.

	Normal puberty (n = 4)	Precocious puberty (n = 5)	*p* value
Sex (Female: Male)	1:3	3:2	0.294
Age (year)	13.1 ± 2.3	12.6 ± 2.1	0.462
Bone age (year)	13.5 ± 2.0	15.6 ± 2.0	0.110
Bone age (SDS)	0.4 ± 0.5	2.8 ± 0.7	0.014^*^
Bone age advancement (year)	0.4 ± 0.5	3.0 ± 0.8	0.014^*^
Height (SDS)	0.2 ± 1.0	-0.5 ± 1.0	0.327
Weight (SDS)	-0.1 ± 0.4	0.1 ± 0.6	0.624
BMI (SDS)	0.0 ± 0.7	0.8 ± 0.8	0.325
Predicted adult height (SDS)	1.0 ± 0.5	-1.6 ± 1.1	0.014^*^
Midparental height (SDS)	-0.4 ± 0.8	0.0 ± 0.5	0.806
(P)AHSDS-MPH (SDS)	1.1 ± 1.3	-1.9 ± 0.9	0.014^*^
△Bone age/year	1.3 ± 0.3	1.2 ± 0.4	0.593
FSH (mIU/mL)	2.3 ± 0.8	3.9 ± 1.1	0.142
LH (mIU/mL)	1.1 ± 0.7	3.1 ± 2.4	0.180

*p < 0.05, Bone age advancement: difference of bone age and chorological age; BMI, body mass index; SDS, standard deviation score; FSH, follicle-stimulating hormone; LH, luteinizing hormone. The bold values indicate statistical significance.

MMP was undetectable in all patients before and after ERT. The creatinine-corrected concentrations of six other phthalate metabolites before and after ERT are shown in **Supplementary Figure 1**. MEHP and MECPP increased in most cases after ERT (*p* = 0.038 and *p* = 0.051, respectively; **Supplementary Figures 1D, E**). However, the average levels of phthalate metabolites did not significantly differ between normal and precocious puberty patients.

## Discussion

ERT has prolonged the survival of IOPD patients, then the growth and puberty in long-term survivors of Pompe disease raised concerns due to frequently long-term plastic medical devise exposure. During our longitudinal follow-up, 75% of girls and 40% of boys with Pompe disease on ERT experienced premature onset of puberty. Although the case number is small, the high prevalence of precocious puberty in Pompe patients urged us to explore factors associated with precocious puberty in Pompe disease patients.

In Pompe disease, the impact of puberty has seldom been described previously. Premature pubarche, caused by increased activity of the adrenal glands, was seen in Pompe disease patients on ERT; suggesting that glycogen accumulation may contribute this phenomenon (8). However, premature pubarche was not observed in our study. In contrast, we found that Pompe disease patients on ERT were at high risk of precocious puberty. In the general population, precocity puberty affects 1–5,000 children and has a 10-fold higher prevalence among girls than boys (22). Rapid pubertal progression, characterized by accelerated physical and osseous maturation, reduces adult height potential. In our study, eighty percent of Pompe disease patients with precocious puberty had a significant short adult height.

For the potential etiology of precocious puberty in our Pompe disease patients, no single factor of obesity, exogenous hormone exposure, or daily diet has significant association in our patients. Given that bis(2-ethylhexyl) phthalate (DEHP) was found to be released from PVC-based medical devices (21), whether ERTs are associated with increased phthalate exposure warrants further investigation. Polyvinylchloride, such as di (2-ethylhexyl) phthalate (DEHP), is widely used in our medical devices in procedures of dialysis, coronary bypass, extracorporeal membrane oxygenation (ECMO), blood transfusion, and total parenteral nutrition. The reproductive and developmental toxicity of DEHP has been reported in rats, mice, hamsters, ferrets, and marmosets (23). Based on these studies, DEHP is classified as category 1B for reproductive toxicity by Scientific Committee on Emerging and Newly-Identified Health Risks (SCENIHR) (23). DEHP is a major plasticizer used in medical products, and its first two metabolites were MEHP and MECCP. The urinary DEHP metabolite levels were reported to be higher in girls with premature thelarche or precocious puberty compared to the normal population (12). Other four phthalate metabolites measured in our study have been reported to interfere with endocrine function, but there is no apparent association with precocious puberty (12). A meta-analysis showed that DEHP exposure was a risk factor for precocious puberty, with a pooled odds ratio of 4.09 (12). In a female rats study, Shao et al. found that DEHP upregulated the activity of the IGF-1/PI3K/Akt/mTOR pathway and expression of GnRH in the hypothalamus, which suggests that DEHP might activate hypothalamic GnRH neurons prematurely through the IGF-1 signaling pathway and promote GnRH release, thus leading to female sexual development (24). However, the association between EDC exposure (especially MEHP and its metabolites) and precocious puberty was inconsistent (25, 26). Rais-Bahrami K et al. showed that the follow-ups of adolescents exposed to significant quantities of DEHP in the neonatal period due to ECMO use had no significant adverse effects on their physical growth and pubertal maturity (27). Our results demonstrated the creatinine-correction levels of MEHP and MECCP were increased after ERT in most cases, despite that so-called phthalate free medical device were used. Nevertheless, there were no significant differences in the levels of phthalate metabolites between patients with and without precocious puberty by this cross-sectional measurement. The previous exposures may be underestimated (28). Thus, further studies are needed to confirm the associations among the aforementioned variables.

One possible reason of high prevalent precocious puberty in Pompe patients is premature pulsatile activity of the hypothalamic-pituitary-gonadal (HPG) axis. There was no lesion detected over the hypothalamus pituitary area in our patients. However, it is unclear whether lysosomal glycogen accumulation in the hypothalamus or pituitary gland contributes to the early activation of the HPG axis. Recently, radiological changes to the white matter of the brain were reported in Pompe disease patients, suggesting pathological accumulation of glycogen (29). Previous studies reported that the neurodevelopmental disability in Pompe disease patients is variable (1); and neurodevelopmental disabilities have a 20-fold increased risk of precocious puberty (30). The pathology involving the HPG axis in Pompe disease patients requires further evaluation.

This study had a few limitations, including its cross-sectional design, lack of information on long-term DEHP exposure, and small sample size. In addition, we only checked the first spot urine samples in the early morning on the day and the next day for ERT administration. The half-life of phthalates may vary between individuals. We therefore may not have caught the peak and trough of elimination. Despite these limitations, our study is so far the largest cohort of IOPD patients who were survived to the age of puberty, and without other common etiologies for precocious puberty, such as overweight and immobilization. Awareness regarding puberty and growth is important, and prolonged prospective follow-up of a larger cohort may help elucidate the pathogenicity of precocious puberty in IOPD patients. In the future, we will consider collecting the accumulation doses, conducting the follow-ups earlier, and implementing repeated measurements, as these factors are important for further investigation.

In conclusion, IOPD patients with ERT are at increased risk of precocious puberty, which may compromise their adult height. We observed a transient increase in phthalate metabolite levels before and after ERT, but the impact on early-onset puberty is unclear. Our findings suggest that monitoring the growth and puberty is essential in the care of Pompe disease patients to improve their health outcomes.

## Data availability statement

The original contributions presented in the study are included in the article/**Supplementary Material**. Further inquiries can be directed to the corresponding author.

## Ethics statement

This study was approved by NTUH-REC No: 200703045R. Written informed consent to participate in this study was provided by the participants’ legal guardian/next of kin.

## Author contributions

M-HC, Y-HC, and Y-CT designed the study. M-JT, M-HC, and Y-CT were responsible for data collection and formal analysis. M-JT wrote the original draft, and M-HC, Y-HC, and Y-CT edited the manuscript. All authors approved this manuscript prior to submission.

## Supplementary material

Supplementary Figure 1 The phthalates concentration before and after the enzyme replacement therapy. Before: before ERT; After: after ERT;▲ with dotted line: precocious puberty girl; △ with dotted line: normal puberty girl; ● with solid line: precocious puberty boy; ○ with solid line: normal puberty boy; **(A)** mono-ethyl phthalate (MEP), **(B)** monobutyl phthalate (MBP), **(C)** mono-benzyl phthalate (MBzP), **(D)** mono-2-ethylhexyl phthalate (MEHP), **(E)** mono(2-ethyl-5-carboxypentyl) phthalate (MECPP) and **(F)** mono(carboxy-isononyl) phthalate (MCiNP).
