# Supplementary figures and images for: Precocious puberty in patients with Pompe disease
Source: Front Endocrinol (Lausanne). 2023 Aug 15;14:1150498. doi: 10.3389/fendo.2023.1150498 (PMC10465365; doi:10.3389/fendo.2023.1150498)

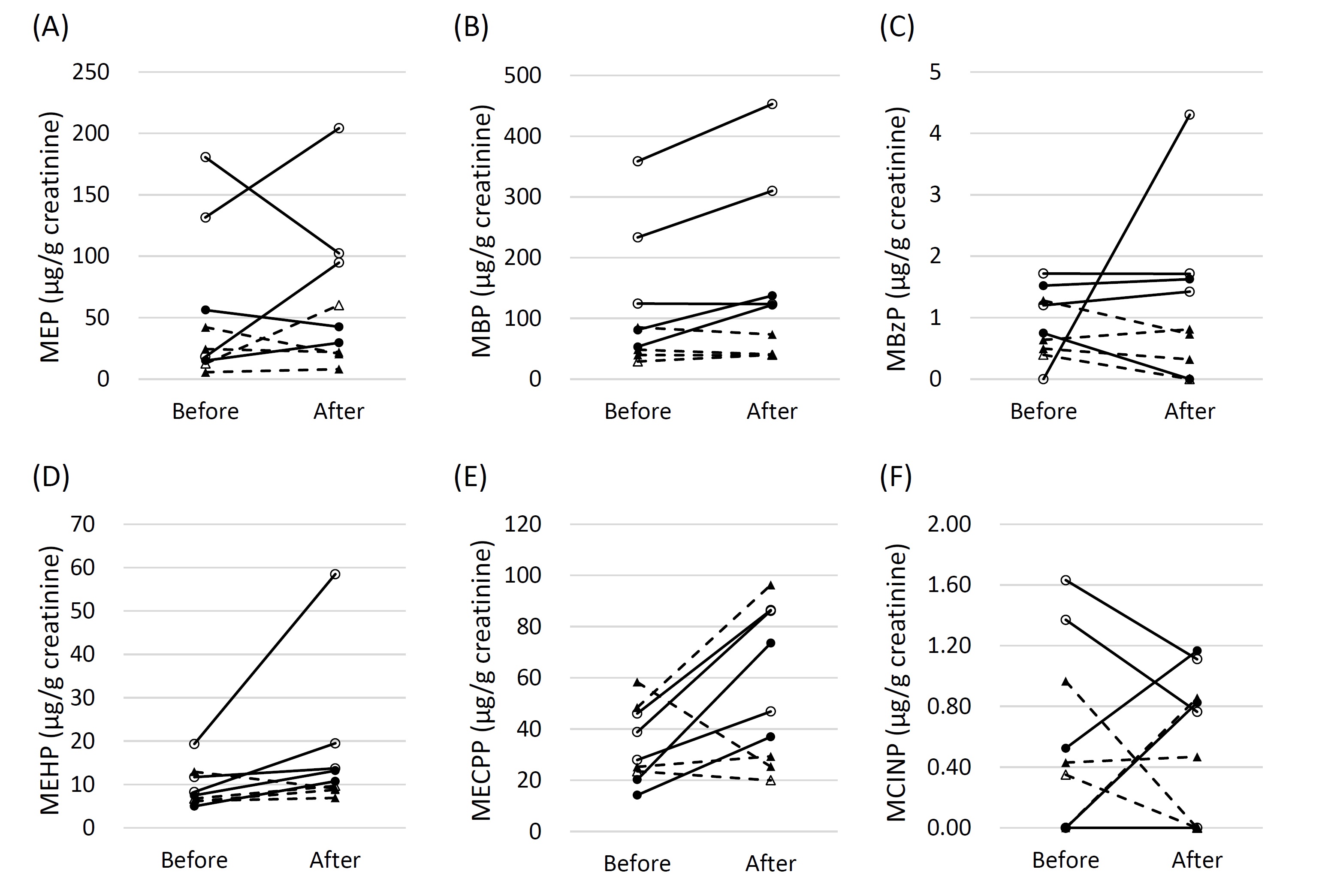

Supplement: Supplementary Figure 1 — The phthalates concentration before and after the enzyme replacement therapy. Before: before ERT; After: after ERT;▲ with dotted line: precocious puberty girl; △ with dotted line: normal puberty girl; ● with solid line: precocious puberty boy; ○ with solid line: normal puberty boy; (A) mono-ethyl phthalate (MEP), (B) monobutyl phthalate (MBP), (C) mono-benzyl phthalate (MBzP), (D) mono-2-ethylhexyl phthalate (MEHP), (E) mono(2-ethyl-5-carboxypentyl) phthalate (MECPP) and (F) mono(carboxy-isononyl) phthalate (MCiNP). [file Image_1.jpg]
